# NetCoDer: A Retransmission Mechanism for WSNs Based on Cooperative Relays and Network Coding

**DOI:** 10.3390/s16060799

**Published:** 2016-05-31

**Authors:** Odilson T. Valle, Carlos Montez, Gustavo Medeiros de Araujo, Francisco Vasques, Ricardo Moraes

**Affiliations:** 1 IFSC—Federal Institute of Santa Catarina, São José 88130-310, Brazil; odilson@ifsc.edu.br; 2 Department of Automation and System, UFSC—Federal University of Santa Catarina, Florianópolis 88040-900, Brazil; carlos.montez@ufsc.br; 3 Department of Computing, UFSC—Federal University of Santa Catarina, Araranguá 88905-120, Brazil; ricardo.moraes@ufsc.br; 4 INEGI—Faculty of Engineering of University of Porto, Porto 4200-465, Portugal; vasques@fe.up.pt

**Keywords:** wireless sensor networks, network coding, linear network coding, reliability, retransmission techniques, cooperative communication

## Abstract

Some of the most difficult problems to deal with when using Wireless Sensor Networks (WSNs) are related to the unreliable nature of communication channels. In this context, the use of cooperative diversity techniques and the application of network coding concepts may be promising solutions to improve the communication reliability. In this paper, we propose the NetCoDer scheme to address this problem. Its design is based on merging cooperative diversity techniques and network coding concepts. We evaluate the effectiveness of the NetCoDer scheme through both an experimental setup with real WSN nodes and a simulation assessment, comparing NetCoDer performance against state-of-the-art TDMA-based (Time Division Multiple Access) retransmission techniques: BlockACK, Master/Slave and Redundant TDMA. The obtained results highlight that the proposed NetCoDer scheme clearly improves the network performance when compared with other retransmission techniques.

## 1. Introduction

Over the last few years, wireless networks are playing an important role in industrial plants, providing a substantial reduction in installation and maintenance costs, and allowing the implementation of more flexible solutions. However, there are some well-known drawbacks to move from wired to wireless networks, especially when both reliable and real-time communications are required.

In order to cope with the reliability and timeliness requirements imposed by industrial applications, a set of new WSN communication standards has been proposed, such as: ISA-100.11a [1], WirelessHART [2] and the amendment IEEE 802.15.4e [3]. There are strong similarities among these standards that adopt TDMA-based MAC (Media Access Control) protocols for real-time communication and use unlicensed frequency bands, whose physical medium is typically susceptible to radio interferences. Within this context, the adequate deployment of sensor nodes is a relevant issue for the system design of WSNs, where connectivity is a basic requirement for the planning and effective operation of wireless networks [4,5].

In complex communication environments, the wireless channel may be affected by electromagnetic noise, by other devices operating in the same frequency band, by fast fading from multipath signal propagation, by shadowing due to temporary obstruction by mobile objects, *etc.* [6]. Typical drawbacks are the loss of connectivity or message corruption and, as a consequence, there will be a loss of data packets, an increase of delay jitter, an increased number of deadline misses and the loss of synchronization among network nodes [7]. Besides, as sensor nodes are generally battery-powered devices, another relevant issue when operating WSNs is related to the reduction of energy consumption of the overall network [8,9].

Some new strategies addressing these drawbacks have been proposed, such as the use of diversity [10]. Two important diversity techniques are temporal and cooperative diversities. Temporal diversity is essentially achieved by sending the same data twice at different instants of time; cooperative diversity is achieved by exploiting the broadcast nature of the wireless medium, where nodes acting as relays create a kind of “virtual array”.

**Figure 1:**
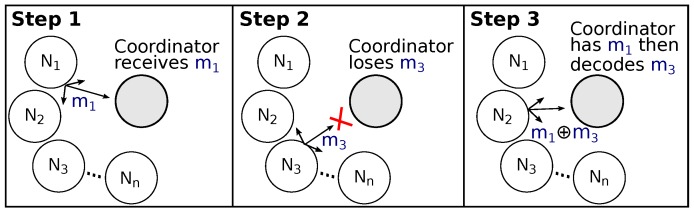
A scenario of using Network Coding to increase the reliability of a wireless star network. Node $N_{2}$ overhears the messages $m_{1}$ and $m_{3}$ and acts as a relay rebroadcasting a single coded message $m_{1}⊕\;m_{3}$.

Network Coding (NC) theory [11] is another promising strategy that has been extensively studied in Internet and wireless networks. Figure 1 highlights a simple scenario combining both cooperative diversity and NC mechanisms in a wireless star-topology network. In this three steps scenario, devices $N_{1}$ and $N_{3}$ want to send data to a coordinator device. In Step 1, device $N_{1}$ broadcasts message $m_{1}$ that is correctly received by the coordinator and that is also overheard by $N_{2}$. In Step 2, device $N_{3}$ broadcasts message $m_{3}$, but $N_{3}$ suffers from destructive interferences and the message is not received by the coordinator. Nevertheless, this message is also overheard by $N_{2}$. In Step 3, device $N_{2}$ that had stored both $m_{1}$ and $m_{3}$ acts as relay and NC encoder, making a simple XOR (exclusive or) operation between them, transmitting a single encoded message. The coordinator recovers the original message $m_{3}$ by applying an XOR operation between the received message ($m_{1}⊕m_{3}$) and the known message $m_{1}$. 

In this communication scenario (Figure 1), there is no feedback from the coordinator to the devices. In this sense, messages received from relays may or may not be useful. Therefore, the number of relays should somehow be dynamically adjusted, considering the impact of realistic PHY (physical) layer and channel conditions. In [12], the authors further discuss this issue, highlighting the relevance of considering the slow fading effect in the design of cooperative MAC protocols. High values of both interference and error rate demand not only more nodes acting as a relay, but also require the use of more complex encoding techniques than simple XOR operations. This is the case of using Linear Network Coding (LNC) theory [13,14], where data obtained from multiple messages is also encoded in a single message. LNC assumes that all messages can be considered vectors of elements from a finite field, and the performed coding can be a linear combination over this finite field. While an XORed NC between two single messages assumes the simplest field of $F_{2^{1}}$ (a binary combination), LNC techniques encoding data from multiple messages require more complex fields (e.g., $F_{2^{4}}$ with 16 combinations or $F_{2^{8}}$ with 256 combinations). Nevertheless, the use of LNC techniques also presents an important challenge: there is a need to send the vector of coefficients used in the linear combination of the message codification.

This paper proposes the NetCoDer mechanism to increase the communication reliability in WSN environments, whose design is based on the merge of the cooperative diversity paradigm with LNC concepts. The NetCoDer behavior has been experimentally assessed using commercial-off-the-shelf (COTS) IEEE 802.15.4 nodes and has also been assessed by simulation, using the OMNeT++ discrete event simulation tool [15].

The main contributions of the NetCoDer scheme are: (i) a novel technique for using NC in WSNs with field size $F_{2^{8}}$, where usually are just used field size $F_{2^{1}}$ due to hardware node constraints; (ii) an original proposal that avoids the overhead of transmitting the intrinsic NC coefficients over the wireless medium; and (iii) a proof-of-concept of implementing this NC technique in COTS WSN nodes.

The paper is structured as follows: Section 2 presents a survey of the state-of-the-art. Section 3 describes the proposed NetCoDer scheme. Section 4 presents the baseline retransmission schemes used for the NetCoDer assessment. Section 5, Section 6 and Section 7 describe the assessment of the proposed NetCoDer technique using both COTS nodes and a simulation tool. Finally, conclusions are presented in Section 8.

## 2. Related Work

The benefits from the usage of NC techniques and relays in WSNs in what concerns energy consumption and reliability represent a well known topic. NC has been proposed to be used at different communication layers, such as physical or medium access control layers. It has also been used in different networking technologies, such as WSNs, traditional wireless networks, Peer-to-Peer networks and many others [16]. This section briefly presents some of the most relevant NC approaches for the MAC layer of IEEE 802.15.4 and IEEE 802.11 networks. Nevertheless, it is worth mentioning that the use of network coding techniques at the physical layer have also been extensively studied [17,18].

In [19], NCCARQ (NC-aided Cooperative Automatic Repeat reQuest) scheme for WSNs is proposed, which is compatible with the non-beacon-enabled mode of IEEE 802.15.4 networks. When implementing this scheme, all the nodes of the network must be operating in promiscuous mode to listen to the ongoing transmissions and cooperate if requested. These packets should be stored until acknowledged by the intended destination. Whenever a packet is not correctly received by its destination, it initiates a cooperation phase by broadcasting a Request for Cooperation (RFC) message instead of an acknowledge (ACK) message. This RFC message is sent after sensing the channel idle for the shortest interframe space. Stations that receive an RFC packet are potential candidates to become active relays. Then, the active relays will try to get access to the channel to persistently transmit the network coded packet. More recently, in [20], this protocol was extended and applied also to IEEE 802.11 networks.

In [21], a MAC protocol for WSNs that acts as relay networks between one or more WBAN (Wireless Body Area Network) to a central process unit (destination) is proposed. This MAC protocol uses random linear network coding techniques, where in the first phase source nodes transmit *N* linear combinations of the *N* original packets. Afterwards, the cloud-assisted control phase starts, where relay actions are coordinated with the help of a cloud manager (centralized coordinator). During this phase, each relay node communicates the sequence number of received packets; the coordinator verifies if all *N* packets have been correctly received. If less than *N* packets have been received, then a retransmission phase takes place: one relay, designated by the cloud manager, transmit requests for retransmission of the missing packets. This process is repeated until all packets are correctly received by the cloud. Finally, the relaying phase starts, in which relays forward the encoded frames to their destinations, according to the schedule issued by the cloud manager.

In [22], the use of NC is exploited to improve the IEEE 802.15.4 performance. Some related practical issues, such as GTS (Guaranteed Time Slot) allocation and multicast, are also discussed in order to efficiently exploit the network coding opportunities. The proposed approach improves throughput and power consumption efficiencies when compared to the original IEEE 802.15.4.

The work presented in [23] COPE (COded PrEamble) applies a linear fountain code to generate encoded packets. At the sender side, it encodes a number of native packets into linear independent packets. At the receiver side, when enough linear encoded packets have been received, the receiver is able to decode the data and obtain the native packets. The main innovation of this approach is the exploration of preambles used to wake up receivers in asynchronous approaches, to encode data packets in wireless sensor networks. COPE encodes multiple native packets and transmits encoded packets as preamble packets to wake up one or multiple receivers. A passive receiver set selection approach is used to let the nodes locally determine whether they receive or not the preamble packets. In the case of unicast or multicast communication, it keeps a set of child nodes. Whenever it receives a packet, it scans the set to determine whether it is in the receiver set of that packet, *i.e.*, if the packet was addressed or not to the node or to one of its child nodes. Receivers send only one ACK packet after receiving enough encoded packets.

A protocol based on relay nodes and Luby codes is proposed in [24], showing that the use of relay nodes can significantly improve the reliability of time constrained data, and reduce the delay in wireless industrial networks. This work analyzes one part of the network composed of three sensor nodes, sending data to the gateway and to a specifically assigned relay node to help these three source nodes. The relay node is always in-between source nodes and the gateway. The performance analysis considers one, two or three time slots allocated for retransmissions, where the relay node first attempts to send the packet belonging to the source with the highest estimated packet error rate, or it sends a Luby coded packet formed from the three source packets. As expected, results conclude that relaying and Luby coding improve performance, mostly when both methods are used in combination. When it is not possible to build a Luby coded packet, it relays the original source packet, one or more times depending on the number of available time slots. The proposed protocol outperforms the ARQ (Automatic Repeat reQuest) protocol commonly used in industrial contexts.

In [25] a new framework was proposed called adaptive network coded cooperation (ANCC). This framework allows the adaptation of network codes to deal with the lossy nature of wireless links and to adapt to the changing network topology. The framework of adaptive behavior captures the nature of wireless medium losses. The proposed framework considerably increases the effectiveness level of cooperation and, consequently, minimizes the total loss rate.

The GDNC—generalization of dynamic-network code—was proposed in [26]. This work increases the diversity order, using non-binary network codes. Moreover, the increase of diversity order aims to minimize the packet error rate on the network. However, the energy and computation costs are not analyzed in the paper.

The work in [25,26] presents NC mechanisms based on the definition of a block of slots. In the first block, nodes broadcast their own messages and, in the second block, nodes relay their partner messages. Within this context, in [27], it is proposed a multi-player game theoretic channel access mechanism for wireless data dissemination, where multiple source nodes aim to balance a tradeoff between saving energy and complete the data dissemination. When applying this mechanism, the base station transmits the information to the “n” nodes in its coverage area in the first block. In the second block, these nodes disseminate the data to the remaining sink nodes. The presented study is focused on the second phase of dissemination, where it uses network coding techniques to assist the progress of the dissemination phase.

In [28], the focus of Girs *et al.* work is the search for the best spatial positioning of relay nodes, aiming for a reliable and timely communication. In [29], it is proposed to use a combination of relaying and packet aggregation at the source nodes. The results show that when relaying and aggregation are used at the source nodes, the transmission schedule plays a crucial role.

In [7], a set of sensor nodes periodically transmits real-time packets to a central controller over lossy links, while using a TDMA-based medium access control protocol. The work assumes the use of relay nodes to assist neighbor nodes with packet retransmissions. The paper focuses on how to schedule TDMA slots for retransmission, using relay nodes in a way that the loss of deadlines is minimized, *i.e.*, it addresses where and how these retransmission slots should be scheduled.

When comparing state-of-the-art approaches that implement network coding to improve the reliability of wireless sensor networks, some significant differences to the NetCoDer scheme can be highlighted. The NetCoDer proposal addresses industrial environments, which are characterized by stable and controlled network topologies. Considering stable network topologies allows the reduction of the complexity associated with the transmission of NC coefficients. Therefore, the NetCoDer scheme is able to minimize the overhead associated with the transmission of NC coefficients to optimize the use of the medium. Moreover, in comparison with [22], the proposed NetCoDer scheme adds cooperative nodes to efficiently retransmit data. Similarly to [28,29], it proposes the use of best link quality as a metric to keep the best node as the relay node. Finally, the target of NetCoDer is the same as [7]. However, the proposed NetCoDer scheme has the distinction of introducing the NC theory, where a set of cooperative nodes relay encoded data, significantly increasing the chances of success for the data transmission.

## 3. NetCoDer Communication Scheme

The rationale behind the NetCoDer scheme is the following: whenever a transmitting node sends data in its assigned slot, there is a set of relay nodes that will later retransmit the correctly received data. Such data will be encoded according to LNC theory, and the resulting coded packet will be transmitted during the retransmission slots. This LNC-based mechanism improves the probability of the data being correctly received at its destination. The number of relays that supports coding and retransmission operations is dynamically adjusted, according to the number of previous unsuccessfully transmitted messages.

### 3.1. System Model

A wireless sensor network for industrial applications typically have multiple sensor devices and one coordinator, generally located at the middle of the topology (star topology). A common way to achieve scalability is to connect multiple star networks, resulting in a star-mesh network. When all sensor devices are routers capable of forwarding packets to and from other sensor devices in the network, it is possible to set up a mesh network topology. Due to the advantages in terms of latency, synchronization, simplicity, predictability and energy efficient behavior, the use of star topologies is suitable for industrial usage [30].

**Figure 2:**
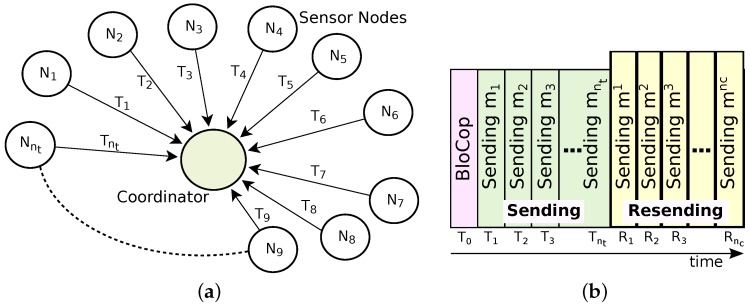
Star topology communication model. (**a**) Messaging model; (**b**) Message slots.

In this paper, we focus on a star topology (Figure 2a), where the coordinator is responsible for the initialization and maintenance of the network, defining its superframe structure. We assume a time slotted medium access scheme, such as the one used in IEEE 802.15.4e, WirelessHART and ISA-100.11a networks [31]. It is well known that, by varying some parameters, it is possible to modify the superframe structure according to the data communication requirements, increasing or decreasing the length of active periods and the duration of the slots.

As shown in Figure 2b, and throughout this text, it will be assumed that there is only one transmission plus one retransmission attempt for each node per beacon interval. The retransmission window is only used by relay nodes. Similar to [24], we also consider systems where no feedback information is available at the relay node, *i.e.*, relay nodes do not know which packets need to be retransmitted. The drawback of not having feedback is that some data in packets will be retransmitted even when it would not be necessary. Decreasing the number of control messages is one of the major advantages of not using explicit feedback mechanisms in WSNs, which is of interest for industrial communication environments.

**Figure 3:**
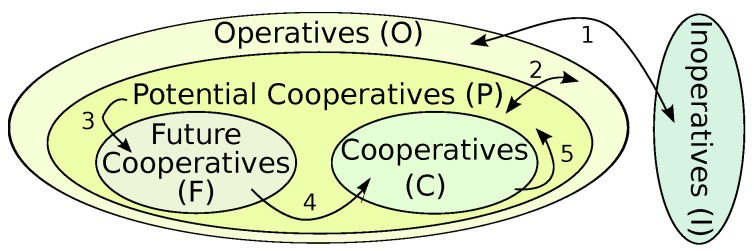
Roles of nodes in NetCoDer.

The NetCoDer scheme considers five subsets of nodes (Figure 3). The *Operative Set* (*O*) is composed of nodes that periodically send messages ($m_{i}$) in their corresponding transmission slots $T_{i}$ (Figure 2b). It is represented by $O=\{N_{1},N_{2},...N_{n_{t}}\}$, where $N_{i}$ identifies the node *i* and $n_{t}$ is the total number of nodes in the network. The *Potential Cooperative Set* (*P*) is composed of $n_{p}$ nodes, pre-selected among the elements of *O*; it defines a subset of nodes that can cooperate among them. The *Cooperative Set* (*C*) is formed by $n_{c}$ relay nodes that, in addition to periodically sending messages, also retransmit coded messages in the retransmission slot ($R_{j}$), where j varies from 1 to $n_{c}$ (Figure 2b). The *Future Cooperative Set* (*F*) is composed of $n_{f}$ nodes pre-selected to cooperate in future cycles. Finally, the *Inoperative Set* (*I*) is formed by nodes registered in the network, but that are not sending messages, or cooperating with any neighbor for some time. A summary of the used notation can be found in Table 1.

The data conveyed in a message $m_{i}$ will have one or more opportunity to reach its destination: in the transmission slot $T_{i}$ or in one or more coded messages ($m^{j}$) sent by one or more relay nodes in retransmission slots $R_{j}$, where $1\leq i\leq n_{t}$ and $n_{c}\leq n_{t}$. Coded message $m^{j}$ is typically a linear combination of several messages successfully listened to, and stored by relay node *i*. Whenever an operative node is also selected to act as relay, it will be included in the relay scheduling list, being able to transmit a coded message in the allocated slot $R_{j}$. In communication scenarios where there is no message losses, no node will be select as relay.

Some of the transitions among the roles of nodes (Figure 3) are as follows:

(1) A node becomes member of set *O* in the setup of the network and it remains inside this set while it keeps sending its own messages. A node is member of *I* if its battery is depleted, or if its links become inoperative, disabling its communication with the coordinator or its cooperative neighbors.
(2) The inclusion of a node in *P* will depend on the algorithm used to select nodes that recently had better transmission quality, based on predefined criteria (e.g., good quality of signal, battery energy power, number of neighbours). Exclusion from this set occurs for the opposite reason.
(3) The number of relay nodes may be dynamically adjusted. The transition of nodes from *P* to *F* may occur due to the need to adjust the number of nodes, usually based on modifications of the network operating conditions.
(4) After a predefined number of cycles, nodes in *F* will migrate to *C* and begin to cooperate within the network.
(5) Finally, after a predefined number of cycles, nodes in *C* will return to *P*, being able to become nodes in *F* again. This operation cycle ensures a fair network behavior in what concerns power consumption.

### 3.2. Detailed NetCoDer Mechanism Description

The first step of NetCoDer consists of determining the $n_{c}$ relay nodes among the $n_{p}$ potential cooperative nodes. This work considers that operative nodes with RSSI (Received Signal Strength Indication) greater than or equal to −87 dBm, measured in the coordinator, are included in the *P* set, as suggested in [32]. An important outcome of [32] is the indication of RSSI as an inexpensive and agile link estimators.

The number of relay nodes ($n_{c}$) in the NetCoDer scheme must be at least equal to the number of message losses in the previous beacon interval. This is in agreement with NC theory, where, by definition, the number of messages must be greater than or equal to the number of variables of the linear system to be solved [33]. The upper bound for the calculation of the number of relays is given by the number of potential cooperative nodes ($n_{p}$). In this calculation, the NetCoDer scheme takes into consideration the log of network operation, counting the number of previous unsuccessfully transmitted messages. It involves the estimation of the number of message losses ($E_{L}$) and its standard deviation ($D_{L}$):

(1) $$n_{c}=min\left(n_{p},\left\lceil \left(\delta \times E_{L}\right)+D_{L}\right\rceil \right)$$

The estimated number of message losses ($E_{L}$) is an exponential moving average based on a weighted combination of the previous value of $E_{L}$ and the new value of $S_{L}$, which is the number of messages that have not been successfully delivered in the previous beacon interval (As the number of slots is equal to the number of nodes with messages to be transmitted, it is expected that the Coordinator receives one message in each transmission slot ($T_{i}$).):

(2) $$E_{L}=\left(1-\alpha \right)\times E_{L}+\alpha \times S_{L}$$

The standard deviation ($D_{L}$) is an estimation of how much $S_{L}$ typically deviates from $E_{L}$:

(3) $$D_{L}=\left(1-\beta \right)\times D_{L}+\beta \times \left|S_{L}-E_{L}\right|$$

The relative weight of $E_{L}$ and $D_{L}$ are determined through *δ*, *α* and *β* coefficients. These parameters will be further discussed in Section 7.

The rationale is to determine the best set of nodes that is able to cooperate in the network, based on a predefined communication quality index ($Q_{i}$). The mean value of two estimators are used for the $Q_{i}$ calculation: the success rate ($H_{i}$) and the normalized link quality indicator ($L_{i}$). The $L_{i}$ value is used mainly because this value has a good correlation with the success rate [32]. Therefore, for each node its communication quality index ($Q_{i}$) is evaluated at the coordinator as:

(4) $$Q_{i}=\frac{H_{i}+L_{i}}{2}$$

where index *i* represents each *P* node, $1\leq i\leq n_{p}$, and $H_{i}$ represents the historical index of successful transmission rates, calculated as:

(5) $$H_{i}=\left(1-\alpha \right)\times H_{i}+\alpha \times S_{R}$$

where $S_{R}=1-S_{L}$.

The coordinator node maintains an ordered list of $Q_{i}$ values for all members of *P*. The $n_{c}$ nodes with higher *Q* value will be elected as relays. The $n_{f}$ subsequent nodes in the list will be inserted in the set *F* of future cooperative relays. For the sake of simplicity, it is considered that $n_{f}=n_{c}$.

### 3.3. Coding Messages

Messages that are sent by cooperative nodes are coded according to network coding (NC) theory. The main theorem of NC theory was proved by Ahlswede *et al.* [11]. Later in 2003 it was proven by Li *et al.* [13] and by Koetter and Medard [14] that LNC is sufficient to achieve reliable multicast transmission at the maximum possible transmission rate in any network. LNC assumes that all messages can be considered vectors of elements from a finite field $F_{q}$ ($m_{i}=\left[m_{i1}\cdots m_{imessagesize}\right],m_{ij}\in F_{q}$), and the performed coding can be as simple as linear combinations over this finite field. To reduce the computational complexity, field $F_{q}$ should be selected as small as possible. Furthermore, all the decoding operations at the receivers can be performed using linear operations.

More precisely, a WSN is modeled as a graph with a set of source nodes. An edge represents a noiseless communication channel of unitary capacity. Assume that there is a set of $n_{t}$ sender nodes organized in star topology and that a subset of these nodes ($n_{c}$) act as relays. Consider that each sender node $N_{i}$ ($i=1\cdots n_{t}$) generates one original message $m_{i}$ per beacon interval and, most probably, listened to by one or more relay nodes. Then, each of the relay nodes will perform a linear combination of the successfully received packets:

(6) $$m^{j}=c_{1}^{i}m_{1}+c_{2}^{i}m_{2}+\cdots +c_{n_{t}}^{i}m_{n_{t}}$$

where $c_{t}^{i}$ is the coefficient of relay node *i* that multiply the incoming message $m_{i}$ received from the neighbor node *t*. If relay node *i* has not been able to hear message $m_{i}$, then $c_{t}^{i}=0$. The coefficients of the linear combination form a vector $c_{t}=\left[c_{1}^{i}c_{2}^{i}\ldots c_{n_{t}}^{i}\right]$, known as coding vector of relay node *i*. More precisely, the coding vector $c_{t}$ associated with relay node *i* is the vector of coefficients of the source symbols that flow (linearly combined) through outgoing edge of relay node *t*.

Consider the final destination coordinator node. *G* the matrix whose *i*-th row is the coding vector of relay node *i*. As soon as the final destination has received $k\leq n_{c}$ linearly independent coded packets, it starts the decoding procedure. Then, the receiver coordinator node has to solve the following system of linear equations:

(7) $$\left[\begin{array}{c} m^{1}\\ m^{2}\\ \vdots \\ m^{k} \end{array}\right]=\left[\begin{array}{cccc} c_{1}^{1} & c_{2}^{1} & \cdots & c_{n_{t}}^{1}\\ c_{1}^{2} & c_{2}^{2} & \cdots & c_{n_{t}}^{2}\\ \vdots & \cdots & & \\ c_{1}^{k} & c_{2}^{k} & \cdots & c_{n_{t}}^{k} \end{array}\right]\left[\begin{array}{c} m_{1}\\ m_{2}\\ \vdots \\ m_{n_{t}} \end{array}\right]$$

(8) $$\left[\begin{array}{c} m^{1}\\ m^{2}\\ \vdots \\ m^{k} \end{array}\right]=G\left[\begin{array}{c} m_{1}\\ m_{2}\\ \vdots \\ m_{n_{t}} \end{array}\right]$$

to retrieve the original packets $m_{i}$, transmitted from the sources and forwarded by relay node.

**Figure 4:**
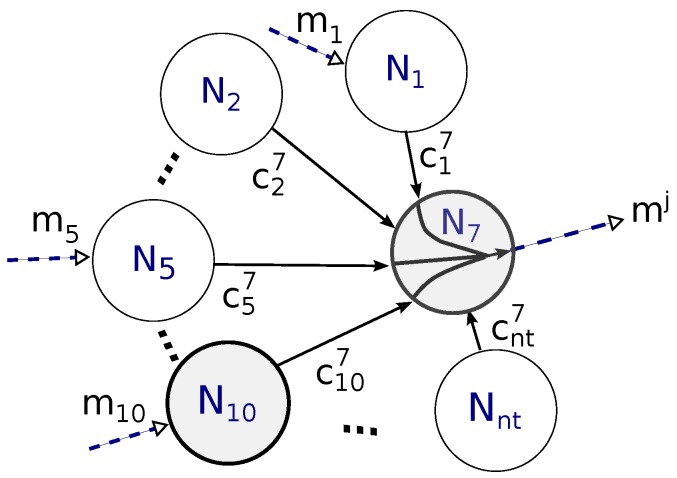
Example of coded message.

Figure 4 illustrates an example where nodes $N_{7}$ and $N_{10}$ (shadowed circles) were selected as relay nodes, depicting the coding coefficients of relay node $N_{7}$, which can potentially listen to messages from $n_{t}$ nodes. The three dotted arrows represent that node $N_{7}$ was able to successfully acquire messages from nodes $N_{1}$, $N_{5}$ and $N_{10}$. Then, the content of coded information retransmit by $N_{7}$ can be derived from the received information, according to Equation (6):

(9) $$m^{j}=c_{1}^{7}m_{1}+c_{5}^{7}m_{5}+c_{7}^{7}m_{7}+c_{10}^{7}m_{10}$$

The coded message $m^{j}$ is then transmitted in the retransmission slot $R_{j}$ (Figure 2b). Retransmission slots are previously allocated to relay nodes by the coordinator. Further details are explained in Section 3.5. In this example, the 7th row of the matrix *G* that would be calculated by the coordinator is:

(10) $$c_{7}=\left[c_{1}^{7}\;0\;0\;0\;c_{5}^{7}\;0\;c_{7}^{7}\;0\;0\;c_{10}^{7}\;\cdots \right]$$

### 3.4. Decreasing the Overhead of Sending Coefficients

Ho *et al.* [34] and Jaggi *et al.* [35] showed that *G* (Equation (8)) would have high probability of being invertible if all the coefficients of all the encoding vectors were randomly selected, independently, and uniformly from the field $F_{q}$, provided that the field size is sufficiently large relatively to the size of the network. Therefore, selecting global coding vectors so that matrix *G* is full rank will enable all receivers to recover the source symbols from the information they receive.

An inherent problem of network coding is the related overhead, given the need to send both the data message and the embedded coefficients used in its coding. We delimited the size of the network to a maximum of 256 nodes and the field size to $F_{2^{8}}$. Given the characteristics of a star topology, we propose the following approach to minimize coding overhead. Each node has an identification in hexadecimal format, representing its position in the slot scale, with addresses ranging from 00 to FF. The coefficient that is used by each node to represent each of its neighbours is obtained from the list of their addresses $N_{0}=00,\;N_{1}=01,\;N_{2}=02,\;\cdots \;,N_{255}=FF$, as represented in Table 2. The rationale is to assign the coefficient $c_{t}^{i}$ based on the address of relay node *i* to neighbour *t*, using the following forming rule: $c_{t}^{i}$=i+tmod256, where *i* is the identification of the *i*-th node in *O* and *t* is the identification of the *t*-th neighbour.

The coordinator is aware of this forming rule and is able to reconstruct the coefficients used in the coded messages. Nevertheless, the coordinator also needs to know which messages each node was able to capture and encode. To fulfill this requirement, each node needs to forward just the addresses of its neighbour nodes, not the entire coefficients. Each relay node *i* sends a sequence of bits that identifies the row in the table, which represents the presence (1) or absence (0) of the message from a node *t* (Table 2).

Considering the example illustrated in Figure 4 for the case of a star network with 16 nodes, it would be necessary to use only the first 16 elements of the first 16 lines. Thus, with node $N_{7}$ encoding messages from neighbours 1, 5, 10 and its own message, it would form a binary number with the following content: 0100010100100000, *i.e.*, it would use only two bytes of overhead. This reduced overhead (from 4 to 2 bytes) should be compared with traditional network coding techniques, where all the used coefficients are sent. For star topologies containing 17–256 nodes, such overhead would vary between 3 and 32 bytes. Compared with 1 to 256 bytes of the conventional method (1 byte for each neighbour), it represents a decrease in the overhead of up to 87.5%.

Finally, it is worth mentioning that the requirement of matrix *G* being full rank, which is equivalent to the requirement that the product of the determinants of *G* needs to be different from zero, was verified to all values of Table 2. Therefore, any set of coefficients that follows this rule could be used as elements of the coding vector on any relay.

### 3.5. Algorithms of Coordinator and Sensor Nodes

**Figure 5:**
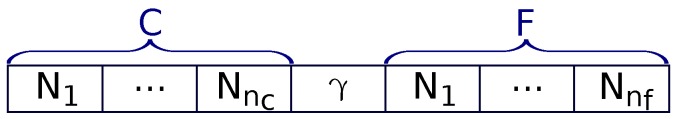
BloCop content.

In NetCoDer, the transmission cycle is synchronized by beacon messages and by a special message called BloCop sent by the coordinator. The BloCop message, as shown in Figure 5, contains: (i) the list of $n_{c}$ current relay nodes (*C*); (ii) a value corresponding to the diversity counter *γ* and (iii) a list of $n_{f}$ nodes that will be selected to cooperate in the future (*F*).

Algorithm 1 illustrates the coordinator behavior. After every *γ* beacon intervals, the coordinator redefines the number $n_{c}$ of relay nodes (redefining the *C* and *F* sets), creating a new BloCop message. The coordinator sends the same BloCop message for *γ* consecutive cycles. Thereafter, in the new BloCop message, if there is no change in the $n_{c}$ value, nodes in *F* will replace nodes in *C* list and set *F* will be filled with the remaining nodes with the higher $Q_{i}$ values. Therefore, the old relay nodes can be removed to the *F* set. If the *C* set does not have $n_{c}$ relay nodes, some of the previous relays can be moved to set *C* again.

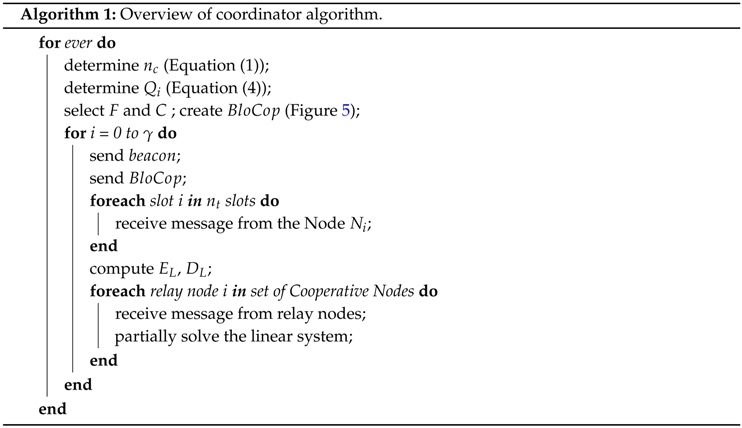


The repetition of *C* and *F* sets in *γ* consecutive BloCop messages increase the temporal diversity and enables the nodes to foresee their future roles. The rationale of this approach is that, in some situations, nodes will continue to collaborate even if they have lost some of the BloCop messages. This operation behavior allows the coordinator to adapt the number of cooperative nodes in accordance with the communication environment. However, this temporal diversity mechanism also reduces the responsiveness of the system. That is, if the network conditions change, the system will take at most *γ* beacon intervals (BI) to react.

Algorithm 2 illustrates the behavior of a sensor node. When a sensor node joins the network, it needs to synchronise itself with both the beacon and the BloCop messages. All $N_{i}$ nodes wait and make a transmission attempt during their respective time slot ($T_{i}$). If node $N_{i}$ is also set as relay, it will stand by listening to and storing all messages sent by all nodes. Afterwards, it will code the successfully received messages (Equation (6)) and wait for its respective slot $R_{j}$ to send message $m^{j}$. The retransmission slots $R_{j}$ are allocated in the same sequence as the node address. Considering the example of Figure 4 ($n_{c}=2$), the order would be $N_{7}$, $N_{10}$. Therefore, node $N_{7}$ act as relay 1 transmitting in the retransmission slot $R_{1}$, sending coded message $m^{1}$. Afterwards, $N_{10}$ acts as relay 2, being able to transmit coded message $m^{2}$ in retransmission slot $R_{2}$. The buffer will be emptied in the beginning of a new beacon interval.

In the NetCoDer scheme, the beacon interval and the slot length are fixed and configured at the system setup time, according to the application requirements. Each node knows the available number of slots ($T_{i}$ and $R_{j}$), which are allocated in the same sequence as the node address.

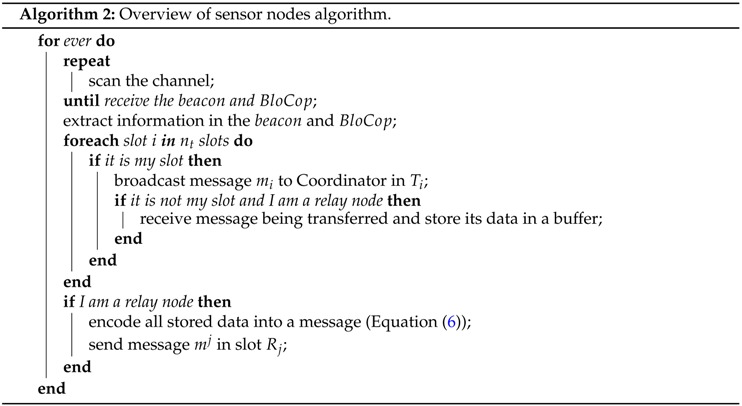


The nodes sleeping time is not explicitly shown in the algorithms. It is assumed that any node that is not executing any relevant activity goes to sleep in order to reduce its power consumption [36].

## 4. Baseline Retransmission Schemes

**Figure 6:**
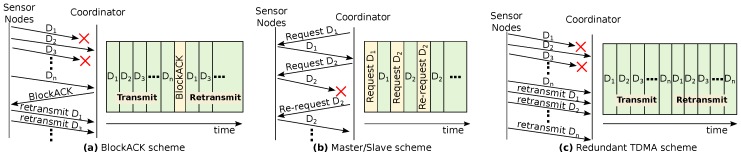
Slotted communication schemes used to increase network reliability. (**a**) BlockACK; (**b**) Master/Slave; (**c**) Redundant TDMA.

Both the simulation and the experimental assessments of NetCoDer were made against a set of three other retransmission communication schemes: BlockACK, Master/Slave and Redundant/TDMA (R-TDMA). All these are TDMA-based techniques designed to enable retransmission opportunities. Figure 6a–c illustrate a timeline of the related message exchanges, the medium occupation and slots used for sending or resending messages. In the case of basic TDMA, there are no retransmission opportunities.

In the BlockACK scheme, Figure 6a, the TDMA frame length is split in two. In the first part, each node has its own slot to send data to the coordinator. The BlockACK frame is a message broadcasted by the coordinator, which contains one bit per node in the data field. If the coordinator has correctly received the message from the respective node, this bit is set to one (ACK); otherwise, it is set to zero (NACK). In the second part of the TDMA frame, retransmission opportunities will be allocated by the coordinator to nodes that received a NACK. Each node, upon receipt of BlockACK and based on its position in the cycle, knows in which slot it will be allowed to retransmit. If there are no lost messages, the frame have just one slot per node, plus one. In the worst case, the frame will contain a number of slots that is equal to the double of operative nodes, plus one.

The use of a BlockACK scheme may be advantageous. Firstly, when compared to the individual ACK sending, it results in a smaller energy consumption and medium occupation. It provides greater temporal diversity, since it distributes messages and acknowledgements in time, which can be of interest in industrial environments. Nevertheless, the use of BlockACK may also increase the average delay jitter, and the loss of the BlockACK message may lead to severe impairments.

In the Master/Slave (pooling) scheme (Figure 6b), the coordinator makes explicit data requests to the nodes. Upon request, the node sends a message containing its data. If the coordinator does not receive the message, it may immediately request a retransmission. It should be noted that this communication scheme has no temporal diversity.

In the Redundant TDMA (R-TDMA) scheme (Figure 6c), the frame is divided in two equal TDMA sets, where each node has two slots to transmit, each one within each of the TDMA block. Therefore, a node sends each message twice (temporal diversity). There is no confirmation of message reception; if at least one of the messages is received, the communication will be successfully completed.

## 5. Experimental Evaluation

Both NetCoDer and the above described retransmission schemes were implemented using a set of MicaZ nodes running the OpenZB micro-kernel, which is compliant with the IEEE 802.15.4 standard. One sensor node was powered by a DC power supply, in order to accurately measure its power consumption. More details about the experimental setup have been reported in [37].

A logical star topology was used, consisting of 9 nodes, one being the coordinator node. All frames had 17 bytes in length, including 9 bytes for header and tail (2 for frame control, 1 for sequence number, 4 for address and 2 for frame check sequence) and 8 bytes for payload. According to the IEEE 802.15.4 standard, the superframe structure has been configured with a beacon interval (BI) equal to 1966.1 ms. Each node generates a frame emulating the act of gathering information from the environment, it processes the related information, stores it into a buffer and sends it in both first and second communication opportunities (when available). The duration of each slot was set to 20 ms for all retransmission schemes. During this time frame, the coordinator is able to send different messages to control the network, which depends on the retransmission scheme being assessed. In the Master/Slave technique, within a time slot there are both a request message from the coordinator and a reply message from the sensor node.

The parameter *γ*, introduced in Algorithm 1 to fix the temporal diversity of NetCoDer, was set to 4. This value was experimentally selected and afterwards used in all experiments.

The target of these experiments is twofold: on one hand to evaluate the feasibility of implementing the NetCoDer communication scheme in COTS wireless sensor network nodes, and to assess their ability to implement on-line network coding algorithms. On the other hand, and more importantly, is to assess the performance of each retransmission scheme and to directly compare them with each other and with a baseline TDMA (TDMA is used in several industrial networking standards, such as WirelessHART). Nodes were randomly arranged, with the maximum distance between them of about 1 m. For all the experiments, messages were collected during about 10 min. This time allows a total of about 300 BIs, *i.e.*, a total of about 5000 slots are allocated, not considering specific compartments of the active part of the IEEE 802.15.4 protocol. This amount of data characterizes a relevant statistical sample.

During the experiments, WSN nodes were deployed near each other, minimizing external interferences to the radio signal. In order to evaluate the behavior of the NetCoDer scheme in the presence of interferences, we induce a controlled packet loss ratio that using a semi-Markov error model, which has been demonstrated to provide a fair approximation of fading in industrial environments [38]. More precisely, a “packet discarding module” was implemented in the coordinator to decide when the packet was delivered or not to the NetCoDer application layer. This module is intended to emulate a controlled Packet Error Rate (PER) and, is similar to the use of a directional antenna to interfere with the receiving nodes [39].

The implemented “packet discarding module” considers that the channel is always in one of two states: Good or Bad, with sojourn times $T_{G}$ and $T_{B}$, respectively. In our analysis, we took an approach similar to [40]. Sojourn times in the two states are exponentially distributed, being the values of $T_{G}$ and $T_{B}$ varied to obtain different values of PER. According to this approach, when a node receives a message, it will decide if the message is corrupted or not, according to its state (Good or Bad). Messages received during the Bad state will be discarded. We assume PER ranges from 0% to 50%, and also that any packet can be lost, regardless being from sensor nodes or from the coordinator.

**Figure 7:**
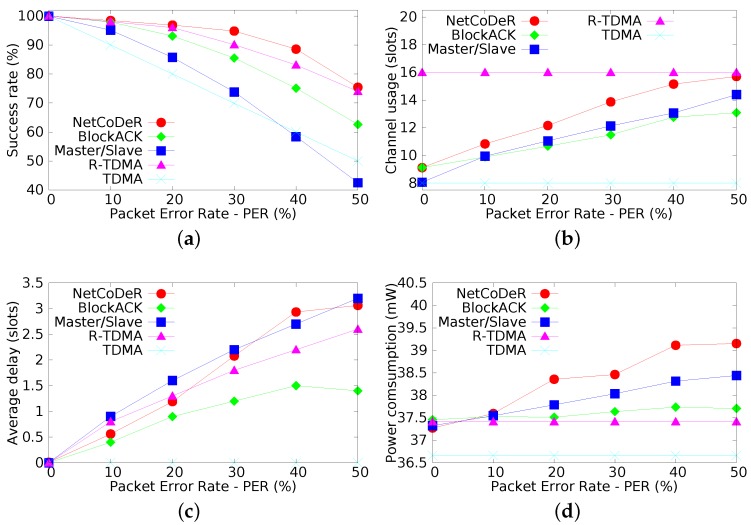
Experimental evaluation. (**a**) Success rate (received/sent data); (**b**) Wireless media utilization; (**c**) Average delay of successfully delivered messages; (**d**) Power consumption of node.

Figure 7a illustrates the data transfer success rate, *i.e.*, the ratio between data sent by sensor nodes and data that actually reaches the coordinator, either in first or in second opportunities. The achieved results highlight that, in general, all techniques have a better performance than the basic TDMA. The NetCoDer scheme has the highest probability of successful transmissions followed by the R-TDMA technique. Therefore, NetCoDer is the technique that ensures the higher reliability to the supported communications.

Figure 7b presents the average of the wireless medium occupancy rate, measured as the number of used slots, according to the PER. This is an important parameter, as the wireless medium is becoming increasingly a scarce resource in industrial environments. As expected, both R-TDMA and TDMA schemes have uniform wireless medium occupancy for any PER level, as they always transmit in both opportunities. The NetCoDeR scheme achieves good performance, keeping a low medium occupancy while ensuring the highest success rate.

Figure 7c presents the average delay of successfully delivered messages (measured in *number of slots*). This metric was obtained by computing the time difference between the first transmission attempt until the successfully deliver of the message at the coordinator (undelivered messages are not considered). In a scenario without errors, the transmission is performed within one slot, and consequently the delay is zero. The experimental results highlight that these techniques have the opposite behavior when compared to the successful delivered rate shown in Figure 7a. This is an expectable result, as more reliable communication schemes take, in average, longer time intervals to successfully deliver messages.

The power consumption of a sensor node is presented in Figure 7d; in Table 3, it is presented an estimation of the lifetime of a node with a 2×2700 mAh battery. In the experiments, the power consumption with the radio turned off was about 22 mW and with the radio turned on about 68 mW [37]. Each result was computed as an average of 550,000 collected samples, representing about 303 macro-cycles. TDMA presents the lowest energy consumption, as the second opportunity transmission has been disabled. The R-TDMA is insensitive to variation of message losses, because the nodes always retransmit the message. Communication schemes with higher reliability have slightly higher power consumption levels, which was also an expectable result.

Summing up, NetCoDer and R-TDMA present the best results in the experimental assessment, specially in what concerns communication reliability. BlockACK and Master/Slave techniques have worse performance ratios, especially in the presence of higher network loss rates. The main reason is because these techniques depend on the adequate delivery of control messages, which are also lost whenever PER increases.

Finally, a natural follow-up question that needs to be answered is: “what is the overhead of a typical WSN COTS node to encode a set of messages?” To answer this question, an additional experimental assessment has been carried out, considering four different types of WSN nodes (Table 4). The experimental assessment included the processing time required to encode 5 messages, varying the message payload length from 8 to 127 bytes.

Table 5 clearly shows that MicaZ is the WSN node type that has the lowest-performance architecture. It takes about 72 ms to encode five 8 byte messages. On the other hand, high performance nodes based on the Atmel WM-400 architecture take only about 1 ms. It is well known that the processing time required to decode messages is significantly higher. However, as the decoding operations are performed by the Coordinator node, it can be implemented using a high performance node.

## 6. Simulation Assessment

A simulation assessment of the NetCoDer scheme was also performed using the OMNeT++ simulator and INETMANET framework. A similar scenario to the one defined for the experimental setup was implemented, but considering a larger number of WSN nodes (30 nodes) placed in random positions, with the coordinator at the central location. For all the simulation experiments, a complete simulation lasted 60 min, during which data was collected, except for the first 40 s, which were excluded to avoid bias in data due to the initial setup.

The INETMANET default parameters for the Battery and PHY parameters were used: battery capacity = 25 mAh, radio power in sleeping mode = 0.06 mA, radio power in idle mod = 1.38 mA, radio power in receiving mode = 9.6 mA, transmitter power = 1 mW, CPU active consumption = 7.6 mA and CPU standby consumption = 0.237 mA.

The ACK were disabled and, variations in PER were simulated with different distances between the nodes. In this regards, the spatial dimensions for the simulation scenario varied from 200 m (0% of losses) to 400 m (∼45% losses) with intervals of 20 m. Differently from the experimental assessment, a frame size of 127 bytes in length, and beacon interval of 3932.16 ms were used.

**Figure 8:**
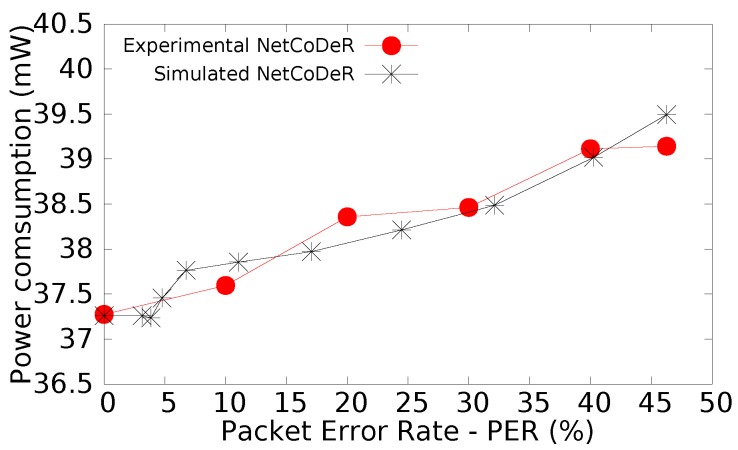
Power consumption of node: Experimental *vs.* Simulated.

Figure 8 presents the power consumption per node comparing the experimental and simulation results, which present quite similar results.

The target of the simulation assessment was to compare the NetCoDer scheme *vs.* the R-TDMA technique, which was the retransmission mechanism that showed better performance in the experimental assessment.

**Figure 9:**
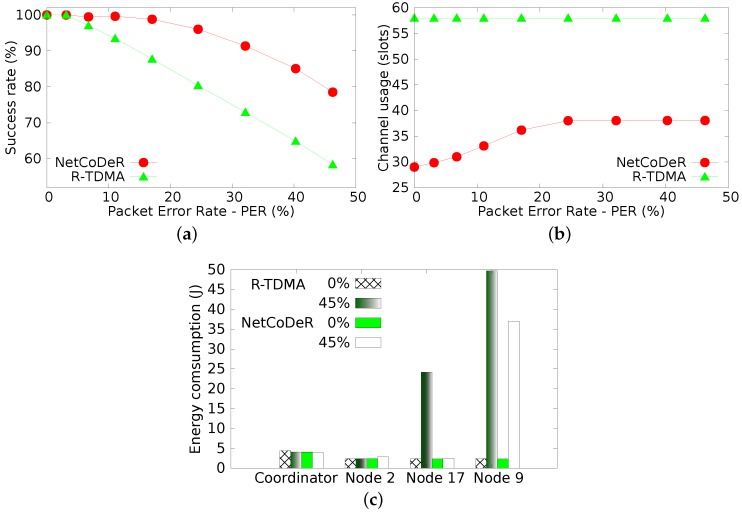
Simulation results. (**a**) Success rate (received/sent data); (**b**) Wireless media utilization; (**c**) Individual battery consumption.

The success rates of both R-TDMA and NetCoDer techniques are shown in Figure 9a. Despite the differences between the considered communication scenarios, namely the higher number of nodes. Simulation results are similar to experimental results shown in Figure 7a. In the simulation assessment both techniques had slightly higher loss rates than in the experimental assessment. This is a consequence of the no beacon losses in the experimental assessment, given the proximity among nodes.

Figure 9b shows the wireless channel utilization, given the number of slots, in relation to the packet error rate. The first observation is that R-TDMA has a uniform medium utilization, regardless of the level of message misses, while NetCoDer has an increasing medium utilization, saturating with PER of about 25%. This saturation is due to the cooperative node selection mechanism (Equation (4)) that is not able to find further cooperating nodes, when the communication error rate is too high (*F* is empty). This characteristic also bounds the success rate of the NetCoDer scheme, as shown in Figure 9a. The performance difference between both techniques also increased. The advantage of the NetCoDer scheme is mainly due to the temporal diversity mechanism, which emulates the auto generation of beacons during up to 4 beacon intervals.

To better understand the individual behaviour of the nodes, Figure 9c illustrates the individual energy consumption for some key nodes in the system: (i) coordinator, which has the same consumption behaviour for both techniques; (ii) node 2, which in both techniques is a node that does not present message losses, and in the NetCoDer technique is always selected as relay node and therefore has a slight increase in energy consumption when the overall message loss rate increases; (iii) node 17, which is a node that presents a medium message loss rate, and, in the case of NetCoDer, remains synchronized to the network and, therefore, has no change in its consumption; however, in the R-TDMA technique, it has successive beacon losses, and thus faces an increase in its energy consumption; (iv) node 9, which is a node that presents a higher message loss rate for both techniques, including the loss of the beacon, and therefore spends more time searching for a beacon, causing an increase of its consumption level. The lower consumption level for the NetCoDer case is due to the intrinsic temporal diversity of this communication scheme.

For both techniques the coordinator significantly decreases its energy consumption at higher PER values. This is due to the fact that the coordinator receives fewer message frames and therefore processes less information.

## 7. Tuning the NetCoDer Parameters

Considering the previous simulation scenario, we were also interested on the impact of setting-up some of the NetCoDer parameters upon its communication behaviour. In this section, we present some results concerning a sensitivity analysis that was performed upon the *γ*, *δ*, *α* and *β* parameters, introduced in Equations (1)–(3) and Algorithm 1.

**Figure 10:**
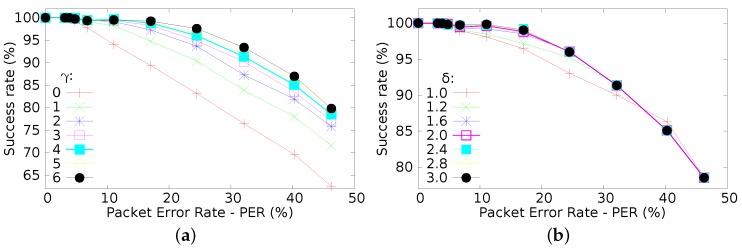
Tuning parameters. (**a**) Effects of varying *γ* parameter; (**b**) Effects of varying *δ* parameter.

Figure 10a presents the success rate when *γ* parameter (diversity index) varies from 0 to 6 (keeping other parameters at the previously defined values). It is clear that *γ* has significant influence upon the achievable success rate. For higher values of *γ*, there is a clear improvement of the success rate of message delivery. This behaviour is mainly due to the emulation of beacon generation for *γ* consecutive times, increasing the tolerance to beacon losses. Therefore, increasing the probability of successfully delivered messages, either when being transmitted in their transmission slots or when being forwarded by cooperative nodes.

A similar sensitivity analysis was performed upon *δ* (Equation (1)). This parameter has a direct influence in the number of cooperative nodes (Figure 10b). A similar improvement can be observed for higher *δ* values, as the number of cooperative nodes increase.

**Figure 11:**
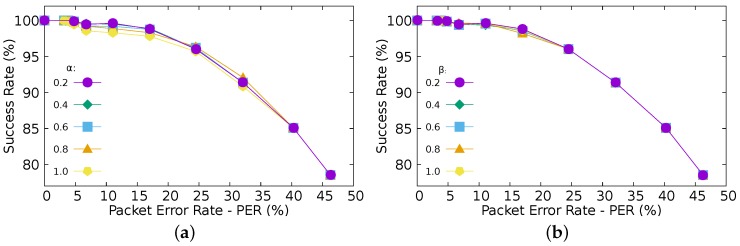
Tuning parameters. (**a**) Effects of varying *α* parameter; (**b**) Effects of varying *β* parameter.

Finally, the sensitivity analysis performed upon *α* and *β* parameters (Equation (2) and Equation (3)) highlights that the effect of considering different weights has a negligible impact upon the estimation of both the number of message losses and the standard deviation (Figure 11).

## 8. Concluding Remarks

In industrial environments, the exchange of information between sensors, controllers and actuators must be carried out in well-defined time intervals, requiring loss-tolerant mechanisms to improve the communication robustness. This paper focuses on the problem of communication reliability in industrial WSNs. It proposes the use of the NetCoDer communication scheme, whose target is to improve the communication reliability and, simultaneously, to reduce the wireless channel utilization by the wireless sensor nodes. When compared to other reliable communication approaches available in the literature, one of the main advantages of NetCoDer is that the number of relays is adjusted according to the network conditions, being required only a set of cooperative nodes operating in promiscuous mode in order to listen to all the ongoing transmissions.

The reliability increase achieved by NetCoDer is due to the usage of cooperative diversity and Linear Network Coding (LNC) techniques applied to the TDMA-based communication paradigm, where some nodes act as relay nodes. These relay nodes use LNC techniques to merge messages from neighbours, creating coded messages that are retransmitted to the coordinator. The LNC techniques assume that all messages can be considered vectors of elements from a finite field $F_{q}$, and the performed coding can be as simple as linear combinations over this finite field. To reduce the computational complexity, field $F_{q}$ should be selected as small as possible. Besides, the NetCoDer communication scheme implements an opportunistic LNC approach, in the sense that the number of cooperative nodes is dynamically adjusted according to the estimation of the link quality.

The main contributions of this paper addresses the use of LNC techniques in real Wireless Sensor Networks: Firstly, it is shown that the field size of the LNC techniques can be as large as $F_{2^{8}}$, in opposition to an usual size of just $F_{2^{1}}$ due to hardware node constraints. Secondly, the proposed NetCoDer scheme avoids the overhead of transmitting the full LNC coefficients through the communication medium; instead, it assigns coefficients based on the addresses of the sensor nodes, being the coordinator able to reconstruct the coefficients used in the coded messages based on the addresses of the sender neighbours. Thirdly, the proposed mechanism uses a reduced number of control messages (BloCop), being these messages repeated, allowing the correct functioning of the network even when they are lost *γ* times, increasing the temporal diversity of the NetCoDer mechanism. Finally, a proof-of-concept was carried out using wireless COTS nodes, demonstrating the feasibility of implementing LNC algorithms upon real nodes.

Through both the experimental and simulation assessments, the NetCoDer communication scheme demonstrated a significantly improved reliability behaviour, when compared with other TDMA-based retransmission techniques. 

## Figures and Tables

**Table 1:** Summary of notation.

Symbol	Meaning
$n_{t}$	Total number of nodes in the network
$n_{c}$	Number of relay nodes
$n_{p}$	Number of potential cooperative nodes
$n_{f}$	Number of future relay nodes
*i*	Transmission index, ranging from 1 to $n_{t}$
*j*	Retransmission index, ranging from 1 to $n_{c}$
*t*	Neighbor index, ranging from 1 to $n_{t}$
$N_{i}$	Identifier of node *i*
$T_{i}$	Transmission slot used by node $N_{i}$
$R_{j}$	Retransmission slot used by relay node *j*
$m_{i}$	Message sent by node $N_{i}$ in transmission slot *i*
$m^{j}$	Coded message sent by relay node *i* in $R_{j}$
$c_{t}^{i}$	Coefficient in hexadecimal based on the address of relay node *i* to neighbor node *t*
$c_{t}$	Coding vector of relay node *i*; $c_{t}=\left[c_{1}^{i}c_{2}^{i}\ldots c_{n_{t}}^{i}\right]$
$Q_{i}$	Quality coefficient of node $N_{i}$
$H_{i}$	Success rate of node $N_{i}$
$L_{i}$	Normalized Link Quality Indicator of node $N_{i}$
*O*, *I*, *P*	Operative, Inoperative and Potential Cooperative node sets
*C*, *F*	Cooperative and Future cooperative node sets
*δ*, *α*, *β*	Coefficients used to tune NetCoDer equations
$E_{L}$	Estimated number of message losses
$D_{L}$	Standard Deviation of $E_{L}$
$S_{L}$	Number of message losses in last beacon interval
$S_{R}$	Complement of $S_{L}$ (that is, 1-SL)
*γ*	Number of consecutive cycles that BloCop message is repeated
*G*	Matrix of the global coding vector
$F_{2^{n}}$	Field in network coding theory with $2^{n}$ symbol combinations

**Table 2:** Hexadecimal coefficients in field size $F_{2^{8}}$.

		Neighbour Address ($t=1\cdots n_{t}$)				
** Node Address** ($i=1\cdots n_{t}$)	00	00	01	02	⋯	FF
	01	01	02	03	⋯	00
	02	02	03	04	⋯	01
	⋮	⋮	⋮	⋮	⋱	⋮
	FF	FF	00	01	⋯	FE

**Table 3:** Estimated lifetime of node (hours) for different PER.

PER	NetCoDer	Master/Slave	Redundant	BlockACK TDMA	TDMA
0%	435	434	433	432	442
50%	414	421	433	430	442

**Table 4:** COTS Nodes architecture.

Platform	Processor	Clock	Flash	SRAM
		(MHz)	(kB)	(kB)
SAM4S-Xplained	ATSAM4SC16	120	1000	128
WM-400	ATSAM4LC4B	48	2560	32
Arduino-Uno	ATMEGA32	16	32	2
MicaZ	ATMEGA128	8	128	4

**Table 5:** Encode times of different nodes.

Platform	Message Size				
	8 Bytes	16 Bytes	32 Bytes	64 Bytes	127 Bytes
SAM4S-Xplained	0.01 ms	0.99 ms	1.00 ms	1.39 ms	2.67 ms
WM-400	0.99 ms	0.99 ms	1.91 ms	3.47 ms	6.77 ms
Arduino-Uno	8.25 ms	16.38 ms	32.61 ms	65.03 ms	129.44 ms
MicaZ	72.32 ms	135.87 ms	272.10 ms	548.05 ms	1125.56 ms
